# Concurrent B-cell acute lymphoblastic leukemia and plasma cell neoplasm with plasmablastic features supporting divergent evolution from a shared precursor: a case report

**DOI:** 10.3389/fonc.2026.1886626

**Published:** 2026-07-10

**Authors:** Antonija Babić, Ivana Franić Šimić, Josip Knežević, Koraljka Gjadrov Kuveždić, Ivana Ilić, Ida Ivek, Lara Divjak, Alojzija Brčić, Klara Bardač, Dora Višnjić, Radovan Vrhovac, Vilma Dembitz, Drago Batinić, Hrvoje Lalić

**Affiliations:** 1 Department of Laboratory Immunology, Clinical Department of Laboratory Diagnostics, University Hospital Centre Zagreb, Zagreb, Croatia; 2 Department of Cytogenetics, Clinical Department of Laboratory Diagnostics, University Hospital Centre Zagreb, Zagreb, Croatia; 3 Department of Pathology and Cytology, University Hospital Centre Zagreb, Zagreb, Croatia; 4 Department of Pathology and Cytology, University Hospital Dubrava, Zagreb, Croatia; 5 Division of Hematology, Department of Internal Medicine, University Hospital Centre Zagreb, Zagreb, Croatia; 6 Department of Physiology and Croatian Institute for Brain Research, University of Zagreb School of Medicine, Zagreb, Croatia; 7 Department of Internal Medicine, University of Zagreb School of Medicine, Zagreb, Croatia

**Keywords:** B-cell acute lymphoblastic leukemia, case report, composite hematologic malignancy, divergent evolution, plasma cell neoplasm, whole-exome sequencing

## Abstract

**Background:**

B-cell malignancies comprise a biologically diverse group of neoplasms, with only rare reports describing the co-occurrence of two distinct lymphoid entities. While concurrent presentation of mature B-cell neoplasms has been documented, the simultaneous occurrence of B-cell acute lymphoblastic leukemia (B-ALL) and plasma cell neoplasm (PCN) has previously been reported rarely. Such cases may provide insight into the clonal architecture and evolutionary pathways underlying lymphoid malignancies.

**Case presentation:**

We report a 76-year-old man presenting with cytopenias and leukocytosis, in whom initial bone marrow evaluation and immunophenotyping established a diagnosis of B-ALL with a pre-B phenotype. At the time of diagnosis, flow cytometry also identified a distinct population of aberrant plasma cells, raising the possibility of a concurrent plasma cell neoplasm. Follow-up bone marrow analysis confirmed the coexistence of two immunophenotypically and morphologically distinct malignant populations: immature lymphoblasts consistent with B-ALL and clonal plasma cells. Initial ALL-directed therapy induced remission of both components. However, subsequent disease evolution demonstrated a clear phenotypic shift, with relapse characterized by expansion of a plasmablastic/plasma cell population consistent with PCN, while the B-ALL component remained undetectable by minimal residual disease assessment. Treatment was therefore redirected toward a myeloma-based regimen, which was limited by toxicity and failed to achieve disease control. Genomic analysis supported the presence of two clonally related but distinct malignancies.

**Conclusion:**

These findings support a model of early clonal divergence from a common progenitor rather than independent malignancies or linear differentiation. This case highlights the complexity of clonal evolution in B-cell malignancies and underscores the importance of integrated genomic and immunophenotypic analysis in understanding disease biology.

## 1 Introduction

Hematologic malignancies of B-cell origin constitute a highly heterogeneous group of neoplasms, encompassing acute and chronic B-cell leukemias, B-cell lymphomas, and plasma cell neoplasms (1). The co-occurrence of two distinct B-cell neoplasms is exceedingly rare but has been reported (2, 3), suggesting a biological potential for polyclonality in these malignancies. Notably, previously published cases have described the co-occurrence of mature B-cell neoplasms—most commonly non-Hodgkin lymphoma—and multiple myeloma (MM). To our knowledge, however, there is only one report describing the simultaneous presentation of B-acute lymphoblastic leukemia (B-ALL) and plasma cell neoplasm (PCN) (4). Additionally, there is one published case where bone marrow aplasia with prominent polyclonal plasmacytosis presented as a prodrome 7 months before the occurrence of B-ALL in a 3 year old child (5). ALL has occasionally been reported as a secondary malignancy following MM therapy, although it occurs far less frequently than therapy-related acute myeloid leukemia or myelodysplastic syndrome (6–8). Whole-exome sequencing of six paired MM and secondary B-ALL samples demonstrated that these malignancies are clonally unrelated, supporting the notion that B-ALL does not arise from de-differentiation of malignant plasma cells, but is a separate neoplasm occurring in response to intensive chemotherapy (9). Conversely, a recently published case study described evolution from B-ALL into smoldering multiple myeloma (SMM) following CD19-targeted CAR-T cell therapy. Longitudinal single cell analyses in that patient showed that it initially presented with highly clonally heterogeneous B-ALL and a small fraction of plasma cell clones. While CAR-T cell therapy successfully eradicated B-ALL, plasma cell clones continued to expand until they presented in the form of PCN (10).

Here, we report a case of simultaneous B-ALL and PCN, with genomic analyses indicating clonally heterogeneous malignancies arising from a shared precursor cell.

## 2 Case presentation

A 76-year-old man was referred to the hematology clinic for evaluation of anemia (hemoglobin 98 g/L), thrombocytopenia (83 × 10^9^/L), and leukocytosis (14.4 × 10^9^/L) with marked lymphocytosis (81%). These abnormalities were initially identified during treatment for right-sided pleuropneumonia. His medical history included arterial hypertension treated with ramipril and a recent deep vein thrombosis managed with rivaroxaban.

**Figure 1:**
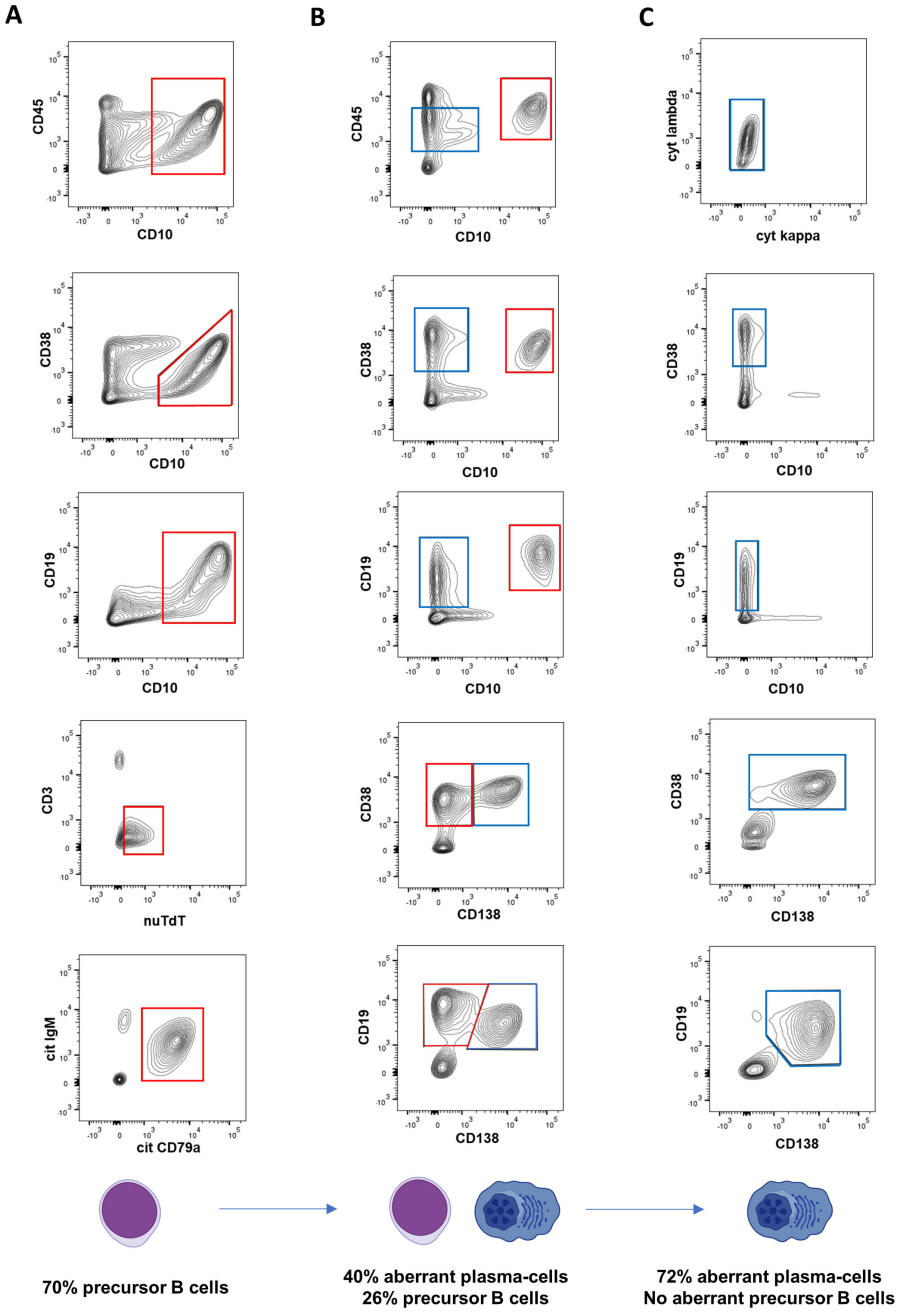
Diagnostic immunophenotyping analyses. **(A)** Initial diagnostic flow cytometry demonstrating pre-B acute lymphoblastic leukemia (B-ALL) with lymphoblasts comprising 70% of bone marrow cells. **(B)** Re-evaluation three weeks after diagnosis identified two dominant populations: residual lymphoblastic leukemia cells comprising up to 26% of analyzed bone marrow cells and approximately 40% aberrant plasma-cell/plasmablastic cells expressing cytoplasmic Ig lambda light chains. **(C)** Relapse assessment showing minimal residual disease (MRD) negativity for the original B-ALL phenotype and expansion of aberrant plasma-cell/plasmablastic population comprising 72% of total bone marrow cells. Immunophenotypic profiles corresponding to lymphoblasts are shown in red, and those corresponding to plasmablasts in blue. Each time point is accompanied by a schematic representation of its immunophenotype.

Based on peripheral blood findings, chronic lymphocytic leukemia was initially suspected. However, bone marrow aspirate cytology revealed extensive lymphoblastic infiltration. Flow cytometric immunophenotyping (**Figure 1A**) demonstrated a predominance of immature B-lineage cells with a pre-B immunophenotype (nuclear TdT+, cytoplasmic CD79a+, cytoplasmic IgM+, surface IgM–, CD10+, CD19+, CD20+, CD34–) and co-expression of myeloid marker CD15, establishing the diagnosis of pre-B acute lymphoblastic leukemia (B-III stage according to EGIL criteria) (11, 12). Fluorescence *in situ* hybridization (FISH) identified a duplication at 1q21 involving the *CKS1B* locus.

**Figure 2:**
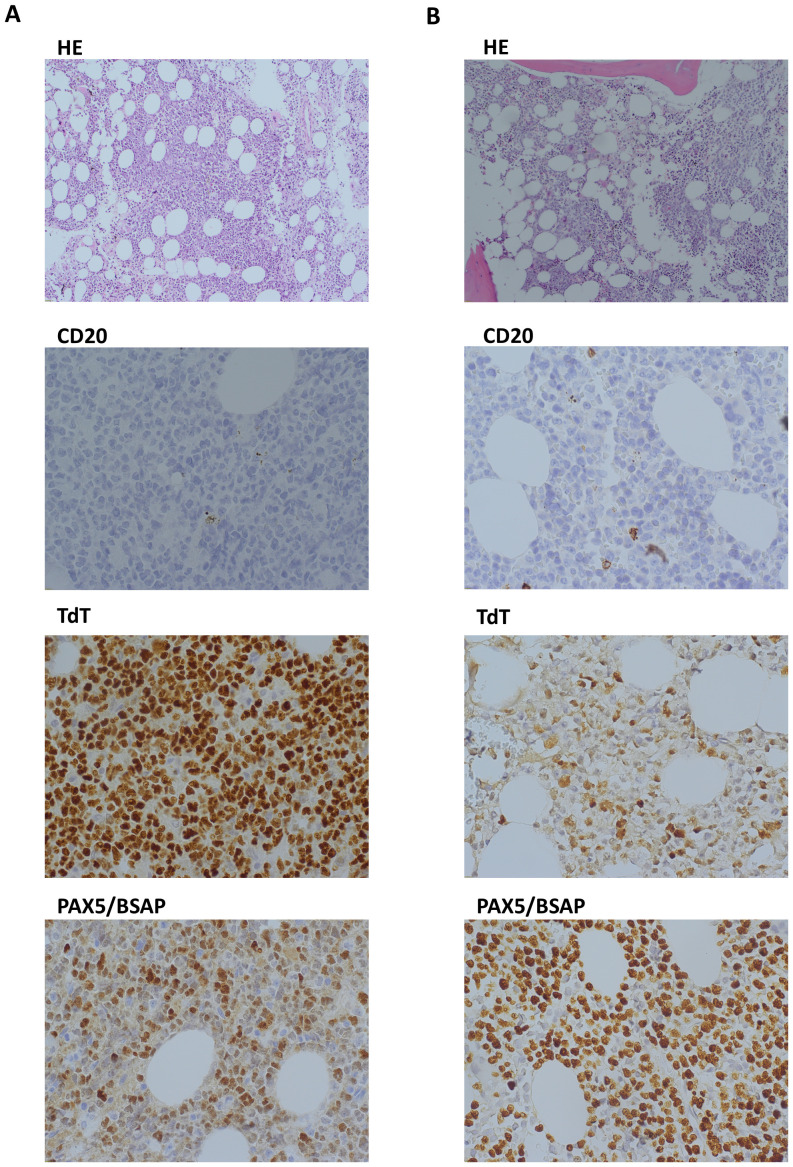
Histopathologic findings. **(A)** Bone marrow morphology obtained three weeks after initial diagnosis of pre-B acute lymphoblastic leukemia (B-ALL), at a time when flow cytometric immunophenotyping demonstrated the presence of both immunoblasts and plasmablasts. **(B)** Bone marrow morphology at relapse, showing extensive infiltration by large atypical cells with immunoblastic/plasmablastic morphology and retained TdT and PAX5 expression. HE - Hematoxylin and eosin staining, TdT - Terminal deoxynucleotidyl transferase, PAX5/BSAP – Paired Box 5/B cell lineage specific activator protein.

Due to insufficient material for histopathological evaluation, a trephine bone marrow biopsy was performed three weeks later, during which time no antileukemic therapy had been administered. Histopathology (**Figure 2A**) showed a 70% cellular bone marrow with extensive tumor infiltration involving approximately 80% of all marrow cells, initially interpreted as lymphoblastic lymphoma/B-cell acute lymphoblastic leukemia based on morphology and immunohistochemistry. Immunohistochemistry showed positivity for TdT and PAX5, while CD20, CD2, CD3, CD5, CD7, CD34, and CD117 were negative. Flow cytometry performed on the bone marrow aspirate identified two dominant aberrant populations: residual leukemic cells comprising up to 26% of analyzed cells and approximately 40% plasma cells expressing cytoplasmic Ig lambda light chains (**Figure 1B**). Serum protein electrophoresis, however, showed no monoclonal gammopathy. The absence of a detectable serum M-protein is compatible with a minimally secretory or non-secretory aberrant plasma-cell population; accordingly, the clonal nature of this population was established by flow cytometric demonstration of cytoplasmic lambda light-chain restriction rather than by serum studies. The detection of a CD10–CD19+ plasma-cell population, not identified at initial evaluation, suggested emergence or expansion of a distinct aberrant plasma-cell clone.

Given the short interval and absence of external triggers, these findings were initially interpreted as spontaneous differentiation of leukemic cells (13, 14). The patient was treated according to an acute lymphoblastic leukemia protocol with vincristine and methylprednisolone; neutropenia was managed with filgrastim.

After four cycles, bone marrow evaluation demonstrated complete cytomorphologic remission with no detectable blasts and minimal residual disease (MRD) of 0.003% by flow cytometry, and no evidence of aberrant plasma cells. Maintenance therapy with mercaptopurine and methotrexate was initiated but discontinued due to hepatotoxicity. During this period, the patient developed bilateral pneumonia, which resolved with broad-spectrum antibiotics and intravenous immunoglobulin for acquired hypogammaglobulinemia.

A subsequent bone marrow assessment two months later confirmed sustained remission with MRD negativity. Maintenance therapy was resumed in an intermittent regimen of nine cycles of vincristine with oral high-dose methylprednisolone. However, one year after initial diagnosis (10 months after remission), follow-up bone marrow evaluation revealed relapse with disease transformation. Cytomorphology showed 65% blast-like cells, while flow cytometry identified 50% aberrant plasma cells and confirmed MRD negativity for the original B-ALL phenotype.

A repeat bone marrow biopsy performed 10 days later demonstrated extensive marrow infiltration, with tumor cells comprising approximately 90% of cellularity (**Figure 2B**). The infiltrate consisted of large atypical cells with immunoblastic/plasmablastic morphology. Immunohistochemistry showed an aberrant B-lineage phenotype with features of plasmablastic/plasma-cell differentiation, including CD138, CD38, CD79a, and LCA positivity, with PAX5 expression and focal MUM1 positivity in up to 50% of cells. The cells were negative for CD20, CD19, CD10, CD34, CD117, CD56, CD99, and HHV8, while TdT was expressed in approximately 60% of cells. *In situ* hybridization for EBV-encoded RNA was negative. Immunohistochemical staining for kappa and lambda light chains did not demonstrate definite light-chain restriction. This finding was discordant with flow cytometry performed at the same timepoint, which clearly demonstrated cytoplasmic lambda light-chain restriction within the aberrant plasma-cell/plasmablastic population (**Figures 1B, C**). Immunohistochemical light-chain restriction assessment is known to lack sensitivity in comparison to flow cytometry and this technical limitation might be an explanation for this discordance (15). *In situ* hybridization for kappa/lambda mRNA was not performed, and clonality of the plasma-cell population was therefore based on the flow cytometric light-chain restriction. Cytogenetic analysis confirmed the previously identified CKS1B duplication.

Although the histopathologic findings raised the possibility of transformation of the original B-ALL clone, repeated flow cytometry demonstrated persistent MRD negativity for the original B-ALL phenotype alongside expansion of an aberrant plasma-cell/plasmablastic population comprising 72% of total bone marrow cells, with an immunophenotype resembling the plasma-cell clone detected at initial evaluation (**Figures 1B, C**). Based on the integrated morphologic, immunophenotypic, cytogenetic, and genomic findings, the relapse was interpreted as progression of the PCN/plasmablastic component rather than recurrence of B-ALL, and treatment was transitioned to a myeloma-based VRd regimen consisting of bortezomib, lenalidomide, and dexamethasone. We emphasize that the relapse population did not correspond to a straightforward, conventional plasma cell myeloma. Rather, its phenotype overlapped both lymphoblastic and plasmablastic/plasma-cell differentiation, retaining TdT and PAX5 expression while acquiring CD138, CD38, and focal MUM1 positivity. This composite, partially immature profile is best regarded as a plasmablastic/plasma-cell neoplasm-like proliferation rather than a typical plasma cell myeloma, and the categorization therefore reflects the dominant biologic phenotype rather than a definitive WHO/ICC disease entity.

Treatment was complicated by severe myelosuppression, including profound neutropenia and thrombocytopenia despite pegylated G-CSF support, necessitating dose reduction and eventual discontinuation of therapy. The patient’s condition progressively deteriorated, with pancytopenia, cachexia, and weakness, culminating in intensive care unit admission for neutropenic septic shock (leukocytes 0.4 × 10^9^/L, platelets 8 × 10^9^/L, C-reactive protein 285.9 mg/L). Despite aggressive supportive care and broad-spectrum antibiotics, he developed respiratory failure, atrial fibrillation, and metabolic acidosis, and ultimately died of refractory septic shock with multiorgan failure.

**Figure 3:**
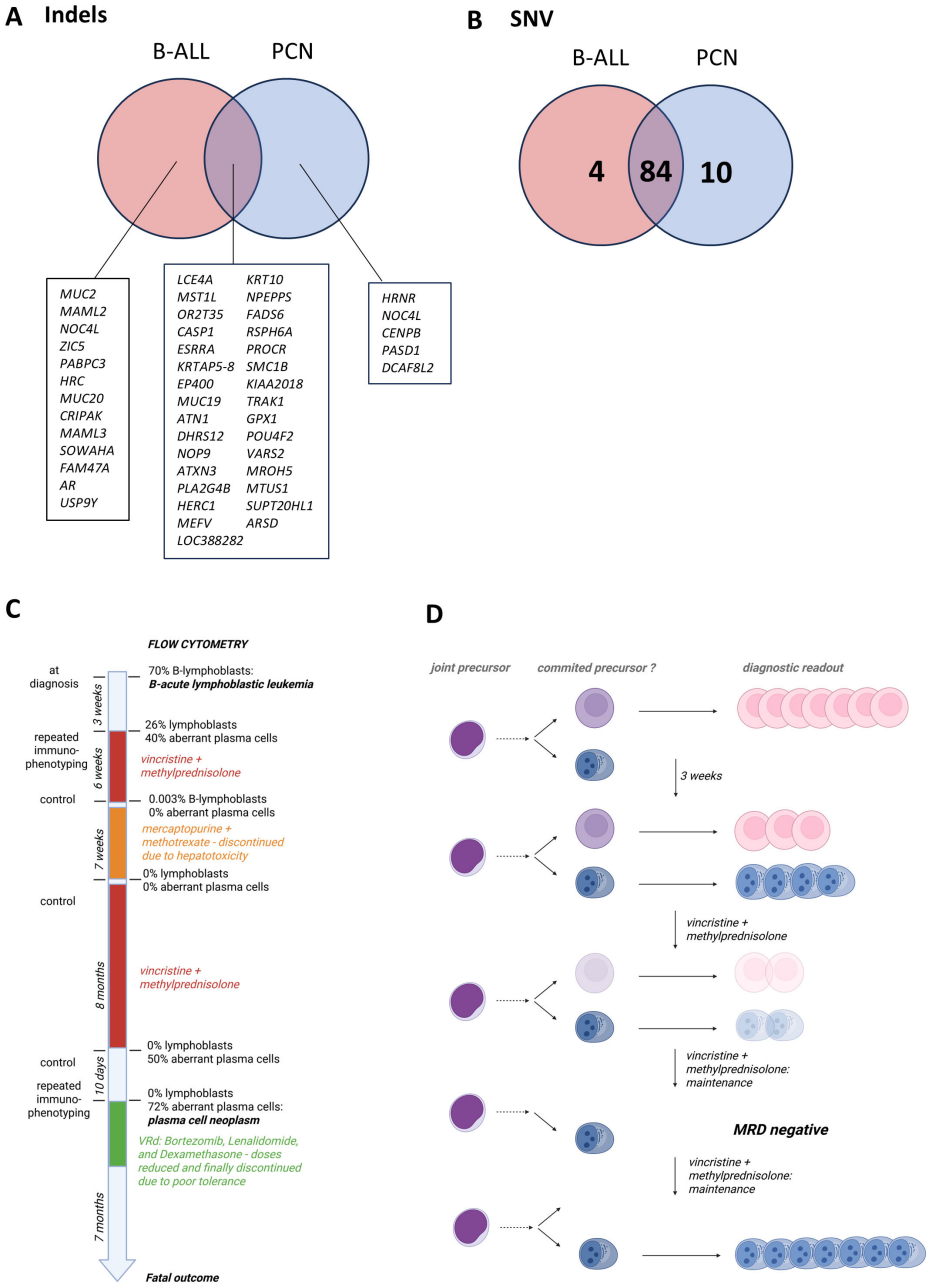
Genetic analyses and disease trajectory. **(A)** Venn diagram illustrating insertions and deletions (indels) identified by whole-exome sequencing in the B-ALL and PCN clones. **(B)** Venn diagram showing the number of single nucleotide variants (SNV) identified by whole-exome sequencing presenting in B-ALL and PCN clones. **(C)** Clinical timeline of disease course, treatment, and relapse. Diagnostic findings are marked in black, while chemotherapeutic protocols are marked in red (B-ALL protocol) or green (PCN protocol). **(D)** Schematic model of clonal evolution and disease progression.

To investigate clonal relationships, whole-exome sequencing (100× depth) was performed on DNA samples obtained at initial presentation (B-ALL clone comprising 70% of marrow cells) and at relapse (plasma cell clone comprising 72%). Variant filtering using the COSMIC database (16) revealed a shared mutational profile alongside clone-specific alterations (**Figures 3A, B**). The absence of B-ALL–specific mutations in the PCN clone argues against direct transformation. Instead, these findings support a model in which both malignancies originated from a common transformed progenitor harboring *CKS1B* duplication and shared mutations, followed by early clonal divergence and acquisition of distinct genetic alterations, resulting in two biologically and clinically distinct lymphoid neoplasms (**Figures 3C, D**).

## 3 Discussion

To date, only a few cases of concurrent B-ALL and plasma cell neoplasm have been reported (4, 10). The co-presentation of two lymphoid malignancies is exceedingly rare; therefore, this case primarily documents an unusual pathological finding rather than providing a basis for broad diagnostic or therapeutic recommendations. The initial ALL-directed treatment was effective in achieving remission of both malignant phenotypes. However, it remains uncertain whether full implementation of a standard myeloma-directed regimen at relapse would have altered the outcome, as treatment was limited by toxicity and the patient’s overall clinical condition. Given the strong CD38 and CD138 expression of the relapse population, anti-CD38-directed therapy (e.g., daratumumab) would be a potential alternative or adjunct to the VRd regimen. However, its use was precluded by the patient’s profound treatment-related cytopenias, intercurrent infections, and rapidly deteriorating performance status, which would have rendered the additional myelosuppression and infection risk unacceptable.

The biological relevance of this case lies in the insight it provides into clonal evolution in lymphoid malignancies, particularly the possibility that distinct B-cell malignancies may arise from a shared precursor. The coexistence of two B-cell malignancies within a single patient raises a fundamental question regarding their clonal relationship—whether they represent independent neoplasms, therapy-related processes, linear transformation, or divergent evolution from a shared progenitor.

Several observations in this case argue against independent or therapy-related malignancies. Genomic analysis demonstrated a shared mutational background between lymphoblastic and plasma-cell/plasmablastic populations, supporting a common clonal origin. This contrasts with previously reported secondary B-ALL after MM, in which paired genomic analyses showed clonally unrelated malignancies (9). Furthermore, both populations were identified early in the disease course, before exposure to cytotoxic therapy, making a treatment-induced process unlikely.

A model of linear differentiation must also be considered. Earlier reports of coexisting B-cell malignancies, such as chronic lymphocytic leukemia and multiple myeloma, suggested maturation of a single malignant clone into a plasma cell neoplasm based on immunologic findings (17). Other reports have suggested transformation across differentiation stages, supported primarily by shared and acquired cytogenetic abnormalities (18). However, such interpretations were based mainly on immunologic or cytogenetic data rather than comprehensive genomic analysis. More recent models of lymphoid evolution indicate that transformation may also occur through divergent evolution from a common progenitor cell, with parallel development of genetically distinct but related malignancies (19). This concept is further supported by genomic studies of composite lymphoid malignancies, which have shown shared early mutations alongside clone-specific alterations in clonally related but phenotypically distinct neoplasms (20).

In our case, the strongest argument against a linear differentiation model is the coexistence of two clearly distinct, immunophenotypically separate malignant populations already at diagnosis—before any cytotoxic therapy—without evidence of intermediate or transitional forms. This pre-therapeutic coexistence indicates that the plasma-cell/plasmablastic population was unlikely to have been generated solely by therapy-driven maturation of the leukemic clone. The persistence of minimal residual disease negativity for the original B-ALL phenotype at relapse is consistent with this interpretation. However, on its own MRD negativity does not fully exclude the possibility that ALL-directed therapy preferentially eliminated the more immature lymphoblastic compartment while permitting expansion of a more differentiated plasmablastic/plasma-cell population. The absence of B-ALL–specific mutational features in the PCN/plasmablastic phase further argues against this population simply representing a differentiated state of the original leukemic clone. Taken together, and weighting most heavily the pre-therapy coexistence of two distinct populations, these observations support a model of early clonal divergence from a common transformed progenitor, resulting in parallel emergence of phenotypically and clinically distinct malignancies.

A further notable feature of this case is the immaturity retained by the relapse population. Despite acquiring plasmablastic/plasma-cell characteristics (CD138, CD38, CD79a, and focal MUM1 positivity), the cells continued to express TdT and PAX5, markers typically associated with immature B-lymphoid differentiation that are not expected in a conventional plasma cell neoplasm. Within the current WHO and ICC frameworks, this phenotype does not map cleanly onto a single defined entity: conventional plasma cell myeloma is characteristically TdT- and PAX5-negative, whereas B-lymphoblastic leukemia/lymphoma lacks plasma-cell terminal differentiation markers. The differential diagnosis of such a lesion includes plasmablastic lymphoma (characteristically EBER-positive and MYC-driven, both absent in our case), B-lymphoblastic leukemia with aberrant plasmacytoid features, and a clonally related plasmablastic/plasma-cell proliferation arising within a composite lymphoid process. The coexistence of TdT and PAX5 with plasma-cell markers most plausibly reflects an aberrant, incompletely differentiated program in a clone that diverged early from a shared precursor, rather than orderly terminal plasma-cell maturation.

This interpretation aligns with contemporary models of clonal evolution in hematologic malignancies, in which early ancestral clones may give rise to multiple subclones that evolve independently under selective pressures (19, 20). The clinical course further supports this model: ALL-directed therapy induced remission of both populations, suggesting at least partial shared treatment sensitivity at an early stage, whereas relapse was characterized by dominance of the PCN/plasmablastic component, reflecting differential treatment sensitivity and clonal selection.

Beyond its clinical rarity, this case provides insight into disease initiation and progression across different stages of B-cell differentiation. In contrast to myeloid malignancies, where leukemia stem cell models and clonal hierarchies have been extensively investigated and are well established in AML and CML (21–23), the concept of a leukemia-initiating cell in B-ALL remains less clearly defined and appears to be more context-dependent. Previous studies have suggested that B-ALL may be propagated either by immature progenitor-like compartments or by blasts across different stages of B-cell maturation, indicating that stem-cell properties in B-ALL may not be restricted to a single immunophenotypically primitive population (24–27). More recent single-cell analyses further support the biological and clinical relevance of HSPC-like blast populations across acute leukemias, including B-ALL, particularly in relation to therapy resistance and clinical outcome (28). In this context, the early emergence of a phenotypically distinct plasma-cell/plasmablastic population, together with shared genomic features, supports the possibility that both disease components arose from an early transformed precursor and subsequently evolved along divergent B-cell differentiation pathways.

This report is limited by its single-case nature. An additional limitation is that clonal relationships were inferred primarily from bulk whole-exome sequencing, without single-cell analyses. Nevertheless, bulk sequencing provided sufficient sensitivity to detect variants with variant allele frequencies above 10% (29). Because the analyzed time points were dominated by either the B-ALL or the PCN/plasmablastic component, as defined by immunophenotyping, this approach enabled comparison of the major genetic features of the dominant malignant population at each stage, while not allowing definitive reconstruction of clonal architecture at single-cell resolution. Accordingly, the proposed model of early clonal divergence should be regarded as the interpretation best supported by the available data rather than as proof.

Finally, this case underscores the diagnostic and therapeutic challenges posed by overlapping lymphoid malignancies and highlights the importance of integrating immunophenotypic, cytogenetic, and genomic data. Although the distinct temporal dominance of each disease component in this case allowed bulk whole-exome sequencing to capture major clonal signatures, higher-resolution approaches will be necessary to more precisely delineate clonal architecture and evolutionary trajectories in composite lymphoid malignancies.

## 4 Conclusion

We report a rare case of simultaneous B-cell acute lymphoblastic leukemia and plasma cell neoplasm, supported by genomic evidence of a shared ancestral clone with subsequent clonal divergence. This observation supports a model in which distinct lymphoid neoplasms may arise in parallel from a common progenitor. The discordant treatment responses further highlight the clinical complexity of such cases, where therapeutically targetable disease components may differ substantially despite a shared origin. Although limited by its single-case nature and the use of bulk sequencing, this report provides insight into the clonal architecture and evolutionary dynamics of B-cell malignancies and underscores the need for further studies—particularly those incorporating single-cell approaches—to better delineate the mechanisms underlying lineage plasticity and disease progression.

## Data Availability

The data presented in this study are deposited in the European Nucleotide Archive (ENA / EMBL-EBI), accession number PRJEB113255. The analysed variant datasets are available in the article’s **Supplementary Material** (**Supplementary Table 1**).
